# Evaluation of efficacy and safety of single and multiple therapy of herbal medicine/Chuna therapy on non-specific chronic low back pain: A study protocol for multicenter, 3-arm, randomized, single blinded, parallel group, incomplete factorial design, pilot study

**DOI:** 10.1097/MD.0000000000021260

**Published:** 2020-07-24

**Authors:** Youme Ko, Bo-Hyoung Jang, Min-Seok Oh, Sun Joong Kim, Yeon-seok Ko, In-Hyuk Ha, Eun Jung Lee, Me-riong Kim, Yun-Kyung Song, Seong-Gyu Ko

**Affiliations:** a Department of Science in Korean Medicine, Graduate School, Kyung Hee University, Seoul; b Department of Korean Rehabilitation Medicine, College of Korean Medicine, Dae-Jeon University, Daejeon; c Department of Korean Rehabilitation Medicine, College of Korean Medicine, Semyung University, Jecheon; d Department of Korean Rehabilitation Medicine, College of Korean Medicine, Woosuk University, Jecheon; e Jaseng Spine and Joint Research Institute, Jaseng Medical Foundation, Seoul; f Jaseng Hospital of Korean Medicine, Seoul; g Department of Korean Rehabilitation Medicine, College of Korean Medicine, Gachon University, Seongnam-si, Republic of Korea.

**Keywords:** chronic low back pain, *Chuna*, herbal medicine, Korean medicine, sogyeonghwalhyeol-tang

## Abstract

**Introduction:**

Chronic non-specific low back pain is one of the common health issues which reduce the quality of life and in working population. While combined therapeutic treatment method is widely used for musculoskeletal related disorders in Korea, well-developed trials on the efficacy of single or combine therapy on herbal medicine and Chuna manual therapy (CMT) are scarce.

**Objective:**

This study aims to evaluate the clinical efficacy and safety of herbal medicine, Sogyeonghwalhyeol-tang (SGHH) on work related chronic low back pain patients. The primary aim is to determine the efficacy of a combined multidisciplinary approach using SGHH with CMT compared to SGHH alone. The secondary aim is to examine the naïve direct comparison between SGHH and placebo.

**Method:**

This trial is designed as a multicenter, randomized, controlled, clinical trial. A total of 150 participants who have with chief complaint of low back pain in Korean medicine rehabilitation center will be randomly assigned to 1 of 3 treatments with a ratio of 1:1:1. Eligible participant will be randomized to treatment arm A receive single treatment of Sogyeonghwalhyeol-tang, in treatment Arm B Sogyeonghwalhyeol-tang and Chuna manual therapy are administered concurrently, in treatment arm C, where individuals receive placebo with Chuna manual therapy. They will receive assigned treatment in 4 weeks and follow-up for 4 weeks. The primary endpoint is to assess the change in severity of low back pain from baseline. The secondary endpoints are the following: the changes in disability and health related quality of life. Adverse events will also be reported.

**Discussion:**

The study result will provide the valuable information for efficacy and safety of monotherapy and multiple therapy of herbal medicinal extract and Chuna manual therapy on chronic non-specific low back pain.

**Trial registration:**

ClinicalTrials.gov Identifier: NCT03132974.

## Abstract 2

Supplemental Digital Content is available in the text

## 1 Introduction

Lower back pain (LBP) is the leading cause of lost productivity in working age adults due to social and economic hardships of medical rehabilitation.^[1]^ Over 80% of adults report LBP, of which nearly 60% develop severe symptoms that last longer due to poor treatment.^[2]^ Most LBPs are non-specific with no identifiable causes, and conventional treatment often targets only pain relief, leading to poor satisfaction.^[3]^

Korean patients suffering from chronic non-specific lower back pain (cnLBP) prefer traditional Korean medicine (TKM), which utilize multiple personalized and holistic treatment methods. The results of the Korean Medical Use and Consumption Survey conducted in 2017 found cnLBP to be the major reason for visits to Korean medical institutions, with a lifetime healthcare services utilization of 52.7%.^[4]^

*Sogyeonghwalhyeol-tang* (SGHH) finds a mention in the *Wanbinghuichun*, a classical treatise on traditional Chinese medicine, where it is indicated for treatment of abnormal musculoskeletal symptoms such as pain, palsy, and stiffness.^[5]^ It is thought that SGHH functions by rejuvenating blood and dredging the meridians and collaterals.^[6]^ The use of SGHH has been reported in several cases of arthritis-induced pain and neurologic symptoms.^[7–9]^

Chuna manual therapy (CMT) is a therapeutic intervention that stabilizes and maintains orthopedic functions by stimulating the meridian system and correcting displacement of the osteoarticular structure.^[10]^ Randomized controlled trials (RCTs) investigating CMT have demonstrated its efficacy in providing relief from musculoskeletal diseases including cnLBP.^[11]^ Though TKM-based monotherapy is common in RCTs for cnLBP, as it avoids complications arising from interaction effects, the holistic nature of TKM demands multiple interventions. Thus, the integration of multiple modalities such as Chinese *Tuina*, Japanese *Shiatsu*, and American chiropractic and osteopathic medicine, may be considered for cnLBP.

Here, we investigate the efficacy of a combination intervention comprising SGHH and CMT for the treatment of cnLBP. While initially, the trial was based on a 2 × 2 factorial design, an arm consisting of a sham CMT with placebo was considered unethical, due to which the trial will be based on an incomplete factorial design (Table 2).^[12]^

The primary outcome of this study is to determine the efficacy of a combined multidisciplinary approach using SGHH with CMT on cnLBP, compared to SGHH alone. The secondary outcome is to compare the efficacy of SGHH and a placebo on cnLBP.

## 2 Methods

### 2.1 Study setting

This study will be a multicenter, assessor- and analyst-blinded, incomplete factorial, randomized, parallel group, placebo-controlled clinical trial. Four university-affiliated TKM hospitals and one private secondary care hospital are chosen to be the study sites – Gil Oriental Medical Hospital of Gachon University, Semyung University Second Affiliated Oriental Medical Hospital at Jecheon, Dunsan Korean Medicine Hospital of Daejeon University Woosuk University of Korean Medicine and the Jaseng Hospital of Korean Medicine. Recruitment will be facilitated using hospital on/offline bulletin boards, and local community-based newspaper advertisements.

### 2.2 Participants

#### 2.2.1 Eligibility criteria

The following conditions will be considered prior to enrollment (Table 1).

**Table 1:**
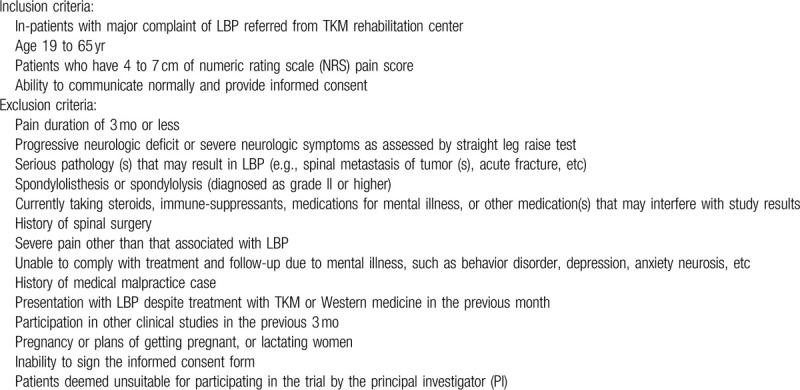
Eligibility criteria.

**Table 2:**
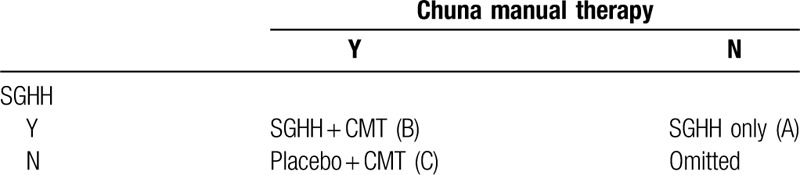
Design of the study groups.

#### 2.2.2 Randomization

**Figure 1:**
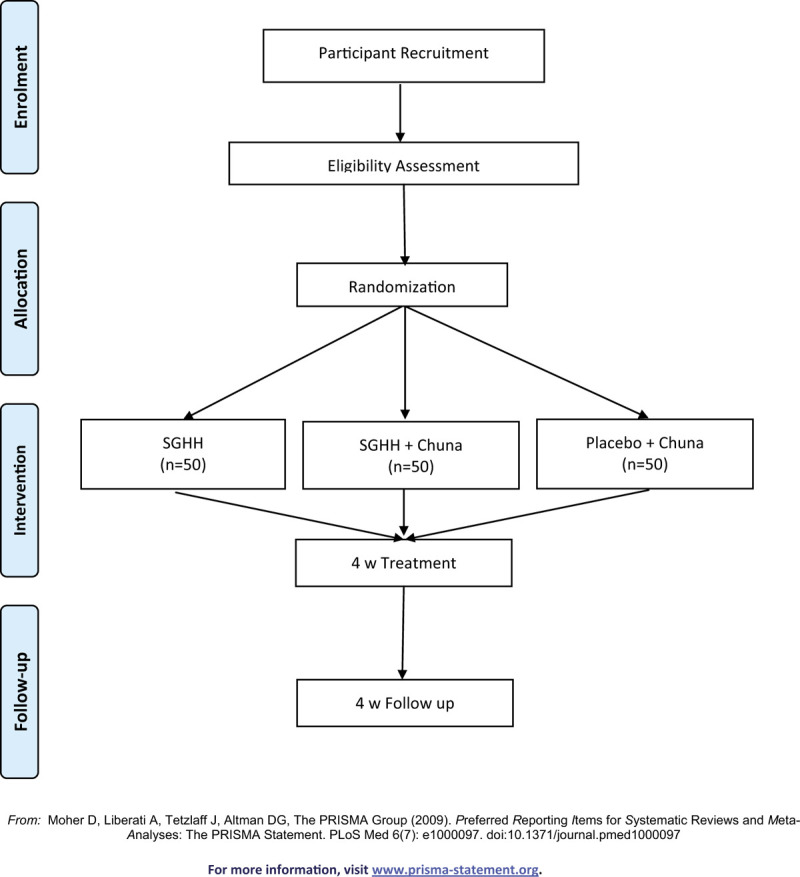
Study flow diagram.

The flow chart describing the trial protocol is shown in Fig. 1. The study population will enroll 150 eligible participants who will undergo a 7-day run-in period prior to randomization. The participants will be randomly allocated to the SGHH group, SGHH with Chuna group or placebo with Chuna group at a ratio of 1:1:1. Random allocation will be facilitated by a web-based application designed by the contract research organization (CRO), the Institute of Safety and Effectiveness Evaluation for Korean Medicine (ISEE), Kyung Hee University. Independent staff from ISEE will use the SAS 6.1 program for randomization list generation and stratification of the trial sites using block sizes of 3 or 6. Once the participant is randomly assigned to a group, patients will not be informed of the type of allocation that they will receive. However, due to diverse intervention strategies, this trial is designed as an assessor- and analyst-blinded study. Participants will be cautioned on confidentiality of allocation prior to outcome assessment and an independent assessor will evaluate allocation in a secured area without seeking information on treatment allocation that the participant will receive. The final dataset will be sent to data analysts without information on treatment allocations to avoid bias in the analysis of study results. In case of expected abnormal symptoms or occurrence of urgent medical conditions, the site's PI will directly inform ISEE and the sponsor to unblind the treatment allocation according to the standard operating procedures (SOPs).

After randomization, the participants will receive medication for 4 weeks, and an additional 4 weeks of follow-up, during which they will visit the clinic once every 2 weeks. To manage adherence to the interventional products, study staff will ask participants to return the unused products for calculating compliance at every visit.

### 2.3 Interventions

#### 2.3.1 SGHH

SGHH is the granulated traditional herbal extract (manufactured by Hanpoong Pharm and Foods Co., Ltd.) for treating musculoskeletal pains caused by arthritis, sciatica, gout, and back discomfort.^[13]^ Subjects will take 3 g of SGHH per serving with warm water, 3 times a day between meals for 4 weeks. The details of ingredients of SGHH are provided in Table 3.

**Table 3:**
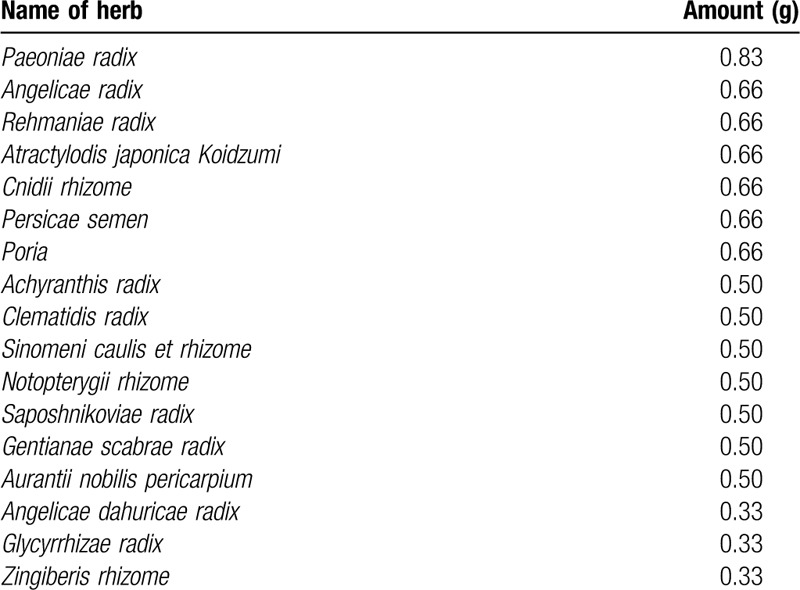
Ingredients details of Sogyeonghwalhyeol-tang.

#### 2.3.2 Placebo

The placebo will be manufactured to appear similar in appearance to SGHH under strict quality-control systems, such that the participants will not distinguish between the 2. The placebo contains 5.1 g lactose, 3.0 g cornstarch, and 0.3 g *Ssanghwa* herbal flavor.

#### 2.3.3 Chuna manual therapy (CMT)

The CMT strategies in this trial are selected after consultation with the scientific committee of the Korean Society of Chuna Manual Medicine for Spine and Nerves (KSCMM).^[14–16]^ Each treatment session conducted by qualified physicians will be 20 to 30 minutes long and will include 3 mandatory and 1 or 2 selective techniques per participant. The CMT physician must be a registered physiatrist of Korean medicine (specialist of physical medicine and rehabilitation) with more than 1 year of clinical experience and should have completed the 126-hour Chuna Manipulative Treatment (CMT) Workshop and have a thorough understanding of study design through an SOP training workshop prior to study initiation. The final selected techniques are presented in detail (Supplemental Digital Content (Appendix 1)).

### 2.4 Withdrawal and dropout

Subjects may withdraw from the trial for any reason, without penalty. If a subject indicates his/her wish to withdraw, the site PI may clarify the reason of withdrawal and discontinue participation. Termination from the study will be at the discretion of the PI in the event of protocol violations such as prohibited concomitant medications and inadequate compliance. All the progress notes on trial terminations are documented on the case report forms (CRF) with reasons.

### 2.5 Outcomes

#### 2.5.1 Primary outcome measurement

We will use numeric rating scale (NRS)^[17]^ as the primary outcome for assessing the severity of LBP, by evaluating intensity of low back pain (LBP) on a scale range of 0 (no pain at all) to 10 (worst pain imaginable). The participants will be asked to rate their pain on a defined scale at each assessment. The changes in the pain intensity between the 3 groups will be analyzed.

#### 2.5.2 Secondary outcome measurement

Secondary outcome measures include evaluation of the functional disability status using Roland Morris Disability Questionnaire (RMDQ),^[18]^ and overall quality of life (QoL) by European QoL 5 Dimension (EQ-5D).^[19,20]^ An adapted Korean version of RMDQ is a reliable measurement for evaluating disability of daily life and consists of 24 questions representing different aspects of functional disability, which is reflected by high total score in the questionnaire. The representative validated QoL measurement tool, EQ-5D, captures an individual's current health status. It contains 3 levels of severity of daily life discomfort which comprises of 5 dimensions, including mobility, self-care, usual activities, pain/discomfort, and anxiety/depression and quantitative self-reported visual analogue scale. The score will be recalculated by Korean estimated weighted-value model of QoL.

#### 2.5.3 Other outcome measurements

Safety outcomes will include physical examination, complete blood count, biochemistry, kidney function test (blood urea nitrogen [BUN] and creatinine), and liver function test (alanine transaminase [ALT], aspartate transaminase [AST], alkaline phosphatase [ALP], and gamma-glutamyl transferase [γ-GT]).^[21]^ Safety will be assessed at screening and visit 6; BUN, creatinine, ALT, AST, and γ-GT will also be assessed at visit 4, and vital signs will be determined at every visit.

#### 2.5.4 Sample size

This is a pilot study to explore the efficacy of single or multiple therapies using SGHH and Chuna, feasibility of the study design and effect size for further large-scale studies. A sample size of 150 patients, which is not estimated based on previous studies, was considered after assessing their LBP clinic visit rates, skilled labor availability, budget, and expected attrition at each site.

#### 2.5.5 Statistical management

An independent statistician from ISEE who is blinded to the treatment allocation will conduct data analysis by both intent-to-treat (ITT) and per-protocol (PP) analysis. The data analysis strategy for testing each study hypotheses is summarized in Table 4.

**Table 4:**
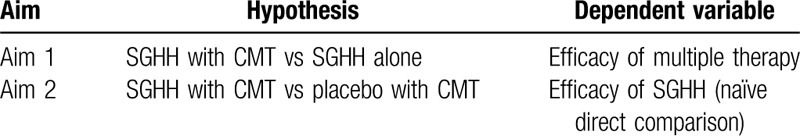
Study hypotheses for statistical analysis plan.

Baseline demographic data will be analysed using a *t* test for continuous data and a chi-square test for categorical data. All outcome measures will be calculated as the mean changes between pre- and post-treatments that will be compared using a paired *t* test for intragroup analysis and a Student *t* test for intergroup analysis. Any variables that are to be assessed repeatedly during the trial will be analysed by comparing the mean changes over time using a repeated-measures ANOVA test. Missing data will be using the Last-observation-carried-forward (LOCF) analysis method. There will be no interim analysis for any reason. And we will not establish the data and safety monitoring committee due to the resource and personnel limitations.

### 2.6 Data monitoring

The ISEE will be responsible for monitoring of collected data and research performance. All study sites will be monitored based on the SOPs during the trial progress. There will be 4 pre-assigned monitoring visits, first enrollment, when 50% of planned enrollment number is achieved, on enrollment completion, and end of trial. During monitoring visits, the clinical research associate (CRA) will review the trial documentation and perform source document verification. After completing data collection, the independent statistician from ISEE will perform data validation and verification by range checks for data values and double data entry for ensuring data quality. And study investigators will have access to the final trial dataset. Any other trial quality assurance committee, such as a coordinating center, a steering committee, an endpoint adjudication committee, etc, will not apply to the current study. No auditing will be performed as well.

### 2.7 Harms

All participants will be informed of potential adverse events (AE) occurring during the trial and prior to enrollment. When AEs occur, the participant may immediately report to the site trial staff. Each AE will be noted in the electronic healthcare record system and CRF by the site trial staff thoroughly and assessed for causality. Serious adverse events (SAEs) will be reported by the site staff to the site principal investigator and the Institutional Review Board (IRB) at the earliest. After receiving an SAE report, the site principal investigator will decide on the continuation of the participant in the trial. If any conditions for compensation to those who suffer harm from trial, we have contracted medical liability insurance for patient safety.

### 2.8 Ethics and dissemination

This trial has been designed in accordance with the amended Declaration of Helsinki and the regulations of the “Good Clinical Practice” principles of the Korea Food and Drug Administration. The protocol has been approved by the Institutional Review Board of 4 participating hospitals – Gil Oriental Medical Hospital of Gachon University, 17-102; Semyung University Second Affiliated Oriental Medical Hospital at Jecheon, 2017-11-04; Dunsan Korean Medicine Hospital of Daejeon University, DJDSKH-17-OR-15, and Oriental Medicine Hospital of Woosuk University, WSOH IRB 1705-01 and Jaseng Hospital of Korean Medicine, Jaseng 2017-08-005). The current version of the protocol is 1.3 and was based on Standard Protocol Items: Recommendations for Interventional Trials (SPIRIT) Statement (see Additional file 1) and consists of all items from the World Health Organization Trial Registration Data Set. All participants will receive detailed explanation and sufficient time to determine trial participation. Hand written informed consent will be obtained from all participants before proceeding with any of the trial procedures. All personal information will be stored in locked cabinets in secured areas in order to protect confidentiality. Important protocol changes must be approved by the IRBs prior to implementation. The results of this study will be published in a scientific journal.

## 3 Discussion

cnLBP affects daily activity and QoL but despite multiple treatment strategies in clinical practice, they have not been systematically evaluated. This protocol was designed to explore the safety and efficacy of SGHH and CMT in patients with cnLBP based on an incomplete factorial design. To the best of our knowledge, this is the first study to evaluate the safety and efficacy of mono- and multiple herbal therapies and CMT on cnLBP.

This study design is not without challenges. First, the results may fail to reach a conclusive conclusion due to the small sample size. Second, incomplete factorial design with naïve comparison may impact the generalizability of the results. Despite these limitations, this will be the first well-planned RCT to investigate the efficacy of SGHH and CMT in cnLBP patients. The findings from this study will provide preliminary efficacy and safety results for future head to head, large-scale RCTs. During the progress of trial, any potential biases that may affect the trial results will be minimized.

Nevertheless, this trial will provide valuable information, such as study design, outcome variables, pain characteristics associated with each occupation, and statistical power for sample size calculation for future large-scale trials and the future development of general clinical guidelines for cnLBP in the field of Korean medicine.

## Author contributions

**Conceptualization:** Min-Seok Oh, Sun Joong Kim, In-Hyuk Ha, Me-riong Kim, Eun Jung Lee, Yun-Kyung Song.

**Methodology:** Youme Ko, Bo-Hyoung Jang, Min-Seok Oh, Sun Joong Kim, In-Hyuk Ha, Eun Jung Lee, Yun-Kyung Song.

**Project administration:** Yun-Kyung Song, Seong-Gyu Ko.

**Statistical consultation:** Bo-Hyoung Jang.

**Supervision:** Yun-Kyung Song, Seong-Gyu Ko.

**Writing – original draft:** Youme Ko.

**Writing – review & editing:** Bo-Hyoung Jang, Min-Seok Oh, Sun Joong Kim, Eun Jung Lee, Yun-Kyung Song.

## Supplementary Material

Supplemental Digital Content
