# Supplementary material for: Evaluation of efficacy and safety of single and multiple therapy of herbal medicine/Chuna therapy on non-specific chronic low back pain: A study protocol for multicenter, 3-arm, randomized, single blinded, parallel group, incomplete factorial design, pilot study
Source: Medicine (Baltimore). 2020 Jul 24;99(30):e21260. doi: 10.1097/MD.0000000000021260 (PMC7387040; doi:10.1097/MD.0000000000021260)
Supplement: Supplemental Digital Content [file medi-99-e21260-s001.docx]

**Appendix 1. Details of Chuna Manual Technique**

| **Mandatory Technique** | |
| --- | --- |
| **Iliopsoas fascial Chuna** | |
|  | Patient lies in the supine position at the caudal end of the Chuna table with the proximal part of the patient’s thighs touching the Chuna table (finding muscle tenderness points, flexing the patient's knees, grasping the knee, bending the hip joint, abduction, external rotation / or internal rotation, and finding a posture where the tenderness points are significantly reduced or mitigated) |
|  | Physician extends the patient’s ipsilateral leg to check the restriction barrier of the iliopsoas muscle/fascia, then backs up to midrange and has the patient breathe in and hold their breath, and implement isometric contraction in the direction of hip flexion using the iliopsoas muscle/fascia (with 20% of maximum force) while the physician applies resistance of the same force. The physician instructs the patient to breath out after 6~7 seconds releasing force, then repeats 3~4 times |
| **Side-lying lumbar flexion dysfunction correction technique** | |
|  | Let patient lies on the side of the rotated side of the vertebra. Then patient's lower elbow is pulled forward, the upper shoulder is positioned rearward, and both arms are positioned on the one side of the trunk for neutral dysfunction and induction of body rotation and lateral flexion in opposite direction. Bend the ankle of the patient's upper leg and hang the ankle on the inside curve of the lower leg. Then, instant correction is performed using the weight of the doctor according to the above correction direction. |
| **Prone iliac posterior rotation/sacral side-bending dysfunction correction technique** | |
|  | Patient lies prone in a relaxed, straight posture on the treatment table. Physician **s**tands contralateral to the patient. The main hand contacts the ipsilateral posterior superior iliac spine (PSIS) with the heel of the cephalad hand of the posterior rotation dysfunctional ilium (posterior inferior ilium). The supporting hand contacts the contralateral ischial tuberosity using the metacarpophalangeal joint (MCP) of the 2^nd^ finger of the caudal hand. |
| **Selective technique** | |
| **Hamstring Relaxation Strengthen Technique** | |
|  | Patient lies prone in a relaxed, straight posture on the treatment table. The affected foot is lowered under the table and the knee is flexed, making the hip joint extended and slightly abducted. Physician face the side of the patient, and finds the tenderness point and reduces the sensitivity of tenderness points by knee flexion and adduction of patella. |
| **Hip Joint Mobilization** | |
|  | Patient lies prone in a relaxed, straight posture on the treatment table. Physician stands by the side of the affected foot and flexes the ankle and knee joint of the patient by 90 degrees, placing the affected knee on the physician’s inner shoulder and holding the anterior thigh with both hands of the physician. Then applying continuous downward traction with joint mobilization. Also, hang the patient's knee over the physician’s neck, and the physician snaps the patient's inner thigh with both hands. Then, applying continuous external traction with joint mobilization. |
